# Bone Marrow Disseminated Tumor Cell Detection Is Beneficial for the Early Finding of Bone Metastasis and Prognosis

**DOI:** 10.3390/diagnostics14151629

**Published:** 2024-07-29

**Authors:** Yulan Wang, Jun Liu, Yanping Gong, Binjie Hu, Jianzhu Xie, Jin Cheng, Qian Huang

**Affiliations:** Cancer Center, Shanghai General Hospital, Shanghai Jiao Tong University School of Medicine, Shanghai 201620, China; wyl_0111@yeah.net (Y.W.);

**Keywords:** disseminated tumor cells, circulating tumor cells, recurrence, metastasis

## Abstract

Background: Disseminated tumor cells (DTCs) are thought to be the initiators of tumor recurrence and metastasis. However, based on the current imaging examination methods, early detection of DTCs is extremely difficult due to their small number and dormant state. Methods: We used the SE-iFISH approach to detect bone marrow DTCs (mDTCs) in patients with breast or prostate cancer, and compared it with various imaging examination methods to explore its role in predicting metastasis and prognosis. Results: Fifteen patients were enrolled in this study. Among them, 11 patients showed imaging-confirmed bone metastases in different sites of the body, of which seven patients had iliac mDTCs and signs of iliac bone metastases on imaging. For the remaining four patients, imaging confirmed that the bone metastatic foci were far from the ilium, but in one patient, mDTCs were detected in the ilium. Interestedly, iliac mDTCs were also detected in two out of four patients who had no sign of bone metastases on imaging. Furthermore, the epithelial marker, CK18, was ubiquitously expressed in mDTCs, but its expression was very low in peripheral circulating tumor cells (pCTCs). The Kaplan–Meier plot suggested that CK18+ mDTCs ≥ 5 was related to poor overall survival (OS) compared with that of CK18+ mDTCs < 5 in breast cancer patients (median OS: 22.1 vs. 46.9 months; log-rank, *p* = 0.035). Conclusions: SE-iFISH examination for mDTCs is more sensitive than the conventional methods used for detecting bone metastases. mDTC detection facilitated the early finding of tumor cells in the bone marrow and ≥5 CK18+ mDTCs was associated with a poor prognosis in breast cancer patients.

## 1. Introduction

Breast cancer ranks first and second in the female incidence and mortality of malignant tumors worldwide. In contrast, incidence and mortality are rapidly increasing in males with prostate cancer [1]. Currently, the treatments for primary tumors are often successful at the early stage, and the occurrence of distant metastasis is usually associated with a poor prognosis. Bone marrow is a common metastatic site for both breast and prostate cancers [2,3]. Inexplicably, distant metastases still occur after curing the primary tumors, which raises questions about their origins. Therefore, exploring the underlying mechanisms is very important for the development of successful cures and improving prognoses.

It is traditionally believed that tumor metastasis often occurs at the late stage, based on the linear model of tumor metastasis: The larger the tumor volume, the greater the risk of metastasis. However, this linear model has been questioned. Cancer cells may have begun to spread and even form metastatic lesions at the early stage, which is called the parallel metastasis model [4]. Tumor metastasis and its subtleties have been puzzling us for a long time, and it has become a difficult problem in tumor recurrence and treatment.

Breast and prostate cancers have a tropism for bones and often show bone metastasis or dissemination [5]. It is known that bone marrow hematopoietic stem cells are usually in a static state. Once the body undergoes an emergency, such as ischemia, these stem cells are activated and produce blood cells to compensate for the loss [6]. Therefore, the specific microenvironment of the bone marrow can provide a natural shelter for tumor cells. Researchers have reported that tumor metastases come from disseminated tumor cells (DTCs) that generally remain dormant to avoid anti-cancer treatments [7,8,9]. Clinically, tumor metastases are often fierce and difficult to cure. Zhang et al. demonstrated that the bone microenvironment invigorates further spread of the disseminated seeds of breast cancer in the bone marrow and that secondary metastases caused by bone marrow DTCs are more difficult to treat than primary tumors in animal models [10]. Therefore, bone marrow disseminated tumor cells (mDTCs) may be the hidden key danger factor for tumor metastasis, which should arouse our attention. Furthermore, it is most noteworthy that, given current imaging methods based on the principle of metabolite detection, disseminated tumor cells are often difficult to detect because they are few in number and often dormant. This may lead to tumor recurrence and the formation of obvious metastatic foci.

Interestingly, we detected numerous mDTCs in bone marrow using the SE-iFISH method in patients with no sign of bone metastases either by bone scan or PET-CT. Therefore, this is enough to question whether tumor cells may already have small metastases when we judge them to have a good prognosis. Moreover, the number of disseminated tiny cell populations may be lower than the detection limit of the current clinical imaging techniques, such as bone scans, PET-CT, CT scans and MRI, which explains why there is no sign of their presence by imaging. Another characteristic of disseminated tumor cells is that they are often dormant and quiescent, which makes metabolism-based imaging detection more difficult. Dormant tumor cells can be activated years or even decades later and cause tumor recurrence and subsequent metastases, which dramatically affect the patient’s prognosis and survival [11]. Grzelak et al. reported that specific lifestyle exposures (e.g., injury, obesity, alcohol, and smoking) may disrupt the microenvironments and lead to the rapid growth of the tumor cells [12]. Therefore, there is an urgent need for a more sensitive and comprehensive method to detect DTCs for earlier detection and treatment of tumor micrometastases.

Due to the heterogeneity of tumor cells, the SE-iFISH method was used in this study to detect mDTCs and circulating tumor cells in peripheral blood (pCTCs). This method combines subtraction enrichment, multicolor immunofluorescence, and FISH technology, and can theoretically detect various types of tumor cells to the greatest extent. As bone marrow is rarely collected from patients with solid cancers, we retrospectively analyzed bone marrow data from only ten patients with breast cancer and five patients with prostate cancer. We found that the SE-iFISH method exposes the inadequacy of conventional imaging in detecting disseminated tumor cells. Although this study has some flaws and limitations due to the small sample size, our primary data suggest that the SE-iFISH method can detect scattered micrometastases in the bone marrow and benefit prognosis and the prediction of recurrence, and therefore deserves to be reported and further investigated.

## 2. Materials and Methods

### 2.1. The Criterion of Patient Enrolment

Patients who had undergone mDTC and pCTC testing at the Cancer Center in Shanghai General Hospital from January 2016 to December 2020 were recruited, including 10 patients with breast cancer and 5 patients with prostate cancer. All patients received an iliac bone marrow puncture for mDTC detection and peripheral blood was drawn for CTC detection on the same day. Patients who received only mDTC or pCTC tests and whose primary tumor was not breast or prostate cancer were excluded. All patients were assessed for bone metastasis by imaging examination, including bone scan, PET-CT, CT scan or MRI, within approximately a week before bone marrow aspiration. The bone scan was done using GE Discovery NM/CT670, and PET-CT by Philips Vereos PET/CT. The bone marrow detection was performed in those patients either with no clear bone metastasis signs based on imaging examination or poor response to treatment after recurrence or progression. Clinical data, including age, gender, operation time, pathological diagnosis, TNM stage, results of sequential imaging examination, amount and trend of serum tumor markers, and treatment protocol were also collected. The detailed procedures for human mDTC and pCTC testing have been previously described [13,14]. Informed consent was obtained from all individuals in this study, and participants consented to have these data and images published.

### 2.2. Detection and Identification of mDTCs and pCTCs by SE-iFISH

The detection of mDTCs and pCTCs was performed using a human peripheral blood circulating tumor cell subtraction enrichment and detection kit (Cytelligen Inc., San Diego, CA, USA), according to the manufacturer’s instructions. Subtraction enrichment (SE) was used for the collection of potential tumor cells in the bone marrow or peripheral blood samples, multiple immunofluorescence staining (i) for the determination or differentiation of tumor markers, and fluorescence in situ hybridization (FISH) for the identification of chromosome alteration cells. The main process was as follows: 6 mL of peripheral blood and 3 mL of bone marrow aspirate were collected using hCTC dedicated sample collection tubes and the samples were processed within 24 h. The first step consisted of centrifugation to obtain karyocytes and remove leukocytes using magnetic beads. The second step involved the utilization of multiple fluorescence dye-conjugated antibodies, including those specific for leukocytes (Alexa Fluor 594-CD45, red) and epithelium-derived cells (Alexa Fluor 488-CK18, green). The third step was to aspirate the stained cells onto a slide and perform in situ hybridization using a chromosome 8-strand-specific probe (Vysis Orange-CEP8, orange) to determine the number of abnormal chromosome 8 cells (S500 ThermoBrite Slide Hybridization System, Abbott Park, IL, USA). The fourth step involved scanning and photography using a Zeiss Fluorescence Microscope and the Metafer Software (Version 3.13.108, Carl Zeiss, Oberkochen, Germany; MetaSystems, Altlussheim, Germany; and Cytelligen, San Diego, CA, USA) to capture various abnormal cells. The fifth step involved a manual evaluation to judge tumor cells in samples. For more detailed steps of the SE-iFISH method, please refer to the Supplementary Materials. It is worth mentioning that we used uniform criteria to identify mDTCs and pCTCs, and all identified cells were reviewed and validated by three experienced, laboratory-certified technicians and pathologists. The criteria to determine whether a cell in the bone marrow or blood was a tumor cell included several aspects, such as cell morphology, size of cell or nucleus, tumor marker staining, and chromosome numbers. There are two types of tumor cells: (1) Tumor marker-negative cells: DAPI^+^/CD45^−^/CK18^−^/chromosome 8 aneuploid cells (triploid, tetraploid or pentaploid and more); and (2) Tumor marker-positive cells: DAPI^+^/CD45^−^/CK18^+^ cells. The cell clusters containing two or more mDTCs or pCTCs were defined as disseminated tumor microemboli (DTM) and circulating tumor microemboli (CTM). The total numbers of DAPI^+^/CD45^−^/CK18^−^/chromosome 8 aneuploid cells and DAPI^+^/CD45^−^/CK18^+^ cells in 6 mL blood or 3 mL bone marrow were set as the pCTC number or mDTC number per patient.

**Figure 1:**
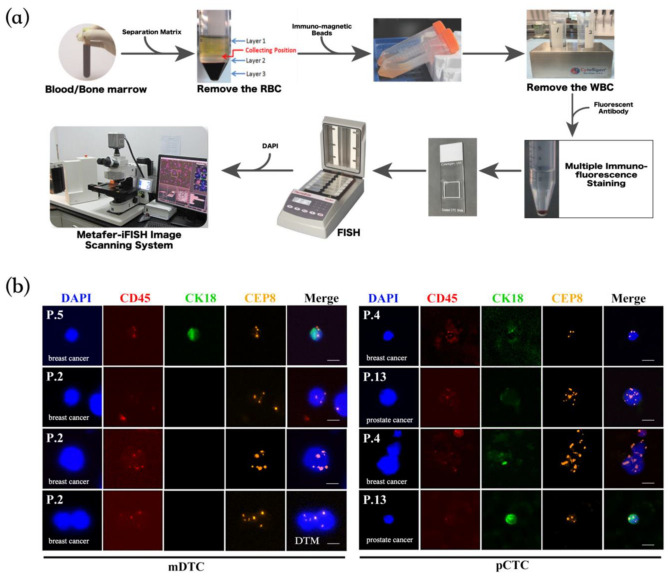
Flow chart of the SE-iFISH method and representative pictures of mDTCs and pCTCs. (**a**) Flow chart of the SE-iFISH method. Firstly, red blood cells were removed with separation solution, then white blood cells were removed with magnetic beads, and finally, multiple immunofluorescence staining and FISH hybridization were performed on the remaining cells. (**b**) Arranged from top to bottom according to the frequency of occurrence of cell subgroups in the bone marrow and peripheral blood. The leftmost part of the pictures is the ID number of the patient from whom the cell was found. The left part shows a representative image of mDTCs and the right part images are the representative images of pCTCs. The blue color represents DAPI staining in the nuclei, the red color represents CD45-positive staining of blood leukocytes, the green color shows the epithelial marker CK18-positive staining, and the orange dots represent the chromosome CEP8. P is the abbreviation for patient. Bars: 10 μm.

It should be noted that due to the spectral overlap between CD45 and CEP8, transmitted light of an orange hue is frequently observed within the red channel. It is established that CD45 and CEP8 are expressed in disparate locations. CD45 is expressed on the cell membrane, whereas CEP8 is situated within the nucleus at chromosomal mitotic binding sites. In such instances, we typically exclude transmission fluorescence based on the site of expression. For instance, the red dotted fluorescence observed in the CD45 channel is typically attributed to the transmission of orange dotted fluorescence from the same site, rather than representing a genuine expression (see Figure 1b CD45 and CEP8 channels for details).

## 3. Statistical Analysis

Continuous variables were expressed as median (interquartile range), and categorical variables were expressed as numbers and percentages. Differences between CK18^+^ and CK18^−^ cells in patients were calculated using a Wilcoxon nonparametric test. The four-table Chi-square test was used to analyze the proportion of patients expressing CK18^+^ cells and CK18^−^ cells. The primary endpoint was overall survival (OS), defined as the time from bone marrow sampling to death due to any cause. Univariate survival analyses were carried out using the Kaplan–Meier method and the Log-rank test for the difference of curve pairs. The reverse Kaplan–Meier method was used to estimate the median follow-up time. *p* < 0.05 indicated a statistical significance. IBM SPSS Statistics 25.0 and GraphPad Prism 8 (Version 8.3.1) were used for statistical analysis.

## 4. Results

### 4.1. General Information of Enrolled Patients

Ten breast cancer patients and five prostate cancer patients were enrolled. All patients’ tumors were primary and had undergone both mDTC and pCTC testing on the same day either before or after treatment. All patients had 1–13 years post-surgery tumor history and had a recurrent or metastatic tumor status, except for patient number 12, who was newly diagnosed with prostate cancer half a month prior. The median age of the patients was 63 years (range: 46–82 years). Among them, patients 9 and 10 were in stage II, and the others were in stage IV. The site of bone marrow aspiration in all patients was the right posterior and superior iliac ridge, and either a bone scan, PET-CT, MRI, or CT scan had been used to determine whether bone metastasis occurred before the bone marrow extraction. Among them, 11 patients had bone metastasis on imaging (seven patients showed iliac bone metastasis, and four patients showed bone metastasis in other sites such as the ribs, spine, femur, humerus, and pelvic bones), and the remaining four patients had no signs of bone metastasis on imaging (Table 1). Although all 15 patients underwent mDTCs examination, only 10 cases were positive (66.7%), of which, seven patients had only CK18^+^ mDTCs, two patients had only CK18^−^ mDTCs, and one patient had both CK18^+^ and CK18^−^ mDTCs. Among the 15 patients, 14 had pCTCs (93.3%), in which three cases (20%) had both CK18^+^ and CK18^−^ pCTCs and the remaining 11 cases had CK18^−^ pCTCs (78.6%). In addition, we also collected the data from relevant serum tumor markers’ testing, such as CEA, CA153, and CA199 (Table 2). The expressions of ER, PR, and HER2 in primary and metastatic sites are also shown in Table 2.

### 4.2. Characteristics of mDTCs and pCTCs

Considering the heterogeneity of tumor cells and epithelial–mesenchymal transition (EMT) during metastasis, we used the SE-iFISH method to capture tumor cells in blood and bone marrow. This method uses a combination of multicolor immunofluorescence and FISH technology, which can simultaneously detect the expressions of several proteins and chromosomal number variations. In addition, negative enrichment can also prevent the loss of tumor cells to the greatest extent. See Figure 1a for more details on the SE-iFISH method. We used the generally expressed epithelial marker CK18 to identify tumor-derived cells and used chromosome 8 probes to detect the changes in the chromosome number. Interestingly, we observed that the mDTCs are mainly diploid CK18^+^ mDTCs and a few are aneuploidy CK18^−^ mDTCs. However, CK18^+^ pCTCs were rarely detected in the peripheral blood, and the majority of pCTCs were CK18^−^ aneuploid pCTCs. It is worth mentioning that we found CD45^−^/CK18^−^ diploid cells, but we did not count them as pCTCs. Typical representative pictures of mDTCs and pCTCs are shown in Figure 1b.

It has been reported that chromosome 8 plays a role in the development and diagnosis of tumors [15,16]. In this study, the copy number of chromosome 8 was enumerated using the CEP8 probe. The ploidy numbers of mDTCs and pCTCs are provided in the Supplementary Materials (see Table S1 for details). Due to the majority of mDTCs displaying diploid (CK18+) status, we did not analyze data related to the number of CEP8 ploidy.

**Figure 2:**
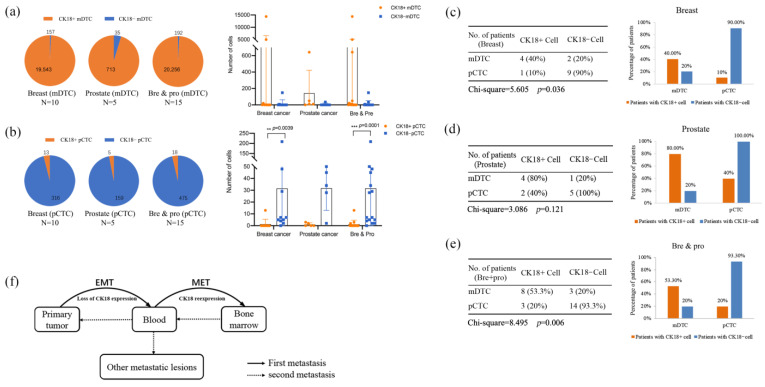
The expression of the epithelial marker CK18 in mDTCs and pCTCs. Pie chart: Counts of CK18^+^ and CK18^−^ cells in mDTCs (**a**) or pCTCs (**b**) in 10 breast cancer patients, 5 prostate cancer patients, and in all patients. The scatter plots show the actual counts in patients. Statistical analysis was performed with Wilcoxon matched-pairs signed rank test. The Chi-square test was performed for the proportion of patients with CK18^+^ or CK18^−^ cells in mDTCs and pCTCs in breast cancer patients (**c**), prostate cancer patients (**d**), and all patients (**e**). The bar chart was added on the right side next to the table chart. (**f**) The diagram of epithelial–mesenchymal transition and mesenchymal-epithelial transition patterns during bone metastases. Bre: breast cancer, Pro: prostate cancer. ** *p* < 0.01, *** *p* < 0.001.

We noticed that the expression of CK18 between mDTCs and pCTCs was different. A total of 19,543 CK18^+^ mDTCs and 157 CK18^−^ mDTCs were detected in the bone marrow of 10 breast cancer patients. Similarly, 713 CK18^+^ mDTCs and 35 CK18^−^ mDTCs were detected in the bone marrow of prostate cancer patients. The scatter plot shows the differential expression of CK18^+^ mDTCs cells and CK18^−^ mDTCs cells in the breast cancer and prostate cancer patients. Unfortunately, the difference was statistically insignificant, probably due to the small sample size and the large range of cell numbers (see the pie chart and scatter plot in Figure 2a for detailed data). In peripheral blood samples, a total of 13 CK18^+^ pCTCs and 316 CK18^−^ pCTCs were detected in breast cancer patients and 5 CK18^+^ pCTCs and 159 CK18^−^ pCTCs were detected in prostate cancer patients. The difference between CK18^+^ pCTCs and CK18^−^ pCTCs in breast cancer patients was statistically significant (*p* =0.0039, see the pie chart and scatter plot in Figure 2b for the detailed data).

Our study further showed that, among the ten breast cancer patients, six had mDTCs, in which four patients were detected CK18^+^ mDTCs and the remaining two patients were detected CK18^−^ mDTCs. In contrast, CK18^−^ pCTCs were detected in nine out of ten breast cancer patients, but one in these nine patients had CK18^+^ pCTCs at the same time (Figure 2c). Consistent with the results of breast cancer patients, four out of five prostate cancer patients had CK18^+^ mDTCs and one in thesefour4 patients had CK18^−^ mDTCs, while all five prostate cancer patients had CK18^−^ pCTCs and only two had CK18^+^ pCTCs (Figure 2d). These differences were statistically significant in the breast cancer patients and the overall population, but not in the prostate cancer patients (Figure 2c–e). The results mentioned above suggested that the blood pCTCs were prone to decreased epithelial marker expression, while the bone marrow mDTCs were prone to recover the expression of epithelial markers, such as CK18. We hypothesized that epithelial-to-mesenchymal transition (EMT) occurs frequently in blood CTCs, while mesenchymal-to-epithelial transition (MET) occurs frequently in bone marrow DTCs (Figure 2f). This is also consistent with the phenomenon that disseminated tumors are often dormant.

### 4.3. Case Analyses of Bone Metastases Detected by SE-iFISH and Traditional Imaging Methods

**Figure 3:**
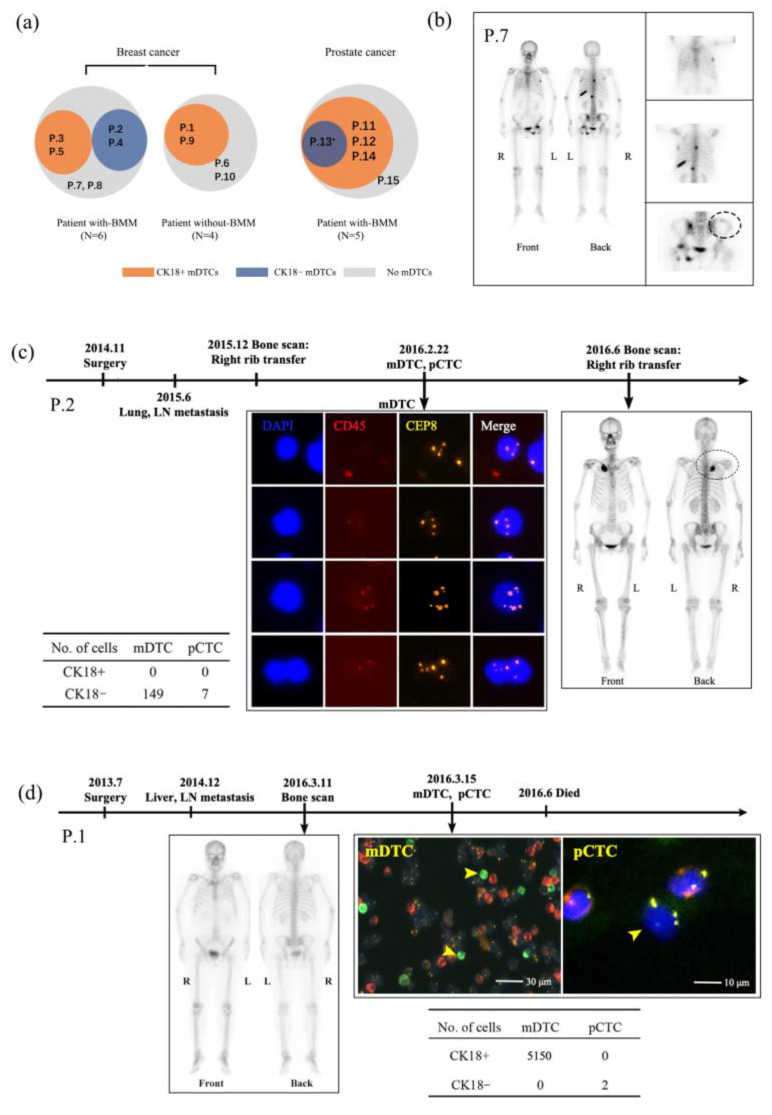
Case analysis of bone metastases through detection by imaging and SE-iFISH methods. (**a**) The diagram of patients with or without mDTCs detected by the SE-iFISH method in breast and prostate cancer patients. The orange color indicates patients with CK18^+^ cells, the blue color indicates patients with CK18^−^ cells, and the gray color indicates patients without mDTCs. (**b**) Case analysis of patient 7: The bone scan showed multiple bone metastases throughout the body, but no metastases in the right posterior and superior ilium. No mDTCs were detected on this site by SE-iFISH. (**c**) Case analysis of patient 2: Two bone scans in half a year did not show metastases in the ilium, but 149 aneuploidy mDTCs were detected in the iliac bone marrow at midway, and 7 aneuploid pCTCs were detected in peripheral blood. (**d**) Case analysis of patient 1: The bone scan showed no iliac metastasis but many mDTCs (5150 tumor cells) were detected in the iliac bone marrow 4 days after imaging examination. Meanwhile, 2 aneuploid pCTCs were detected in the peripheral blood. BMM: bone marrow metastasis by imaging, LN: lymph node, P: patient. * Patient 13 had both CK18^+^ mDTCs and CK18^−^ mDTCs.

We further analyzed the detection efficiency of bone metastases by the SE-iFISH method and compared it with the traditional imaging methods such as bone scans and PET-CT. According to the imaging detection, the patients were divided into two groups: with-BMM (bone marrow metastases) and without-BMM. Among the ten breast cancer patients, six patients showed bone metastases by imaging, in which only four patients were detected as having mDTCs by SE-iFISH, and two patients did not show mDTCs (left Venn diagram in Figure 3a). In the prostate cancer patients, all five patients showed bone metastases on imaging but only four patients showed mDTCs (right Venn diagram in Figure 3a). As to the reason for failing to observe mDTCs by SE-iFISH in these three patients (P.7, P.8, and P.15), it is simply because there was no sign of bone metastasis in the ilium on imaging, such as in patient 7 (Figure 3b). The detailed bone metastases sites that were detected by imaging and mDTC numbers are shown in Table 2. All bone marrow aspirations were performed on the right superior and posticous iliac edges. Furthermore, patient 2 only showed metastases in the right ribs based on the bone scan, but 149 aneuploid CK18^−^ mDTCs were detected in the iliac bone marrow sample (Figure 3c). Patient 1 showed liver and lymph node metastasis rather than bone metastasis on imaging and had a large number of CK18^+^ mDTCs (5150 cells, Figure 3a,d) as detected by SE-iFISH. These results indicated that current clinical imaging examinations cannot fully reflect bone metastasis, especially micrometastasis, which may have caused the loss of the best time for the early identification and intervention for bone metastasis, leading to further spread.

### 4.4. mDTCs Counts Correlated with Patients’ Overall Survival (OS)

**Figure 4:**
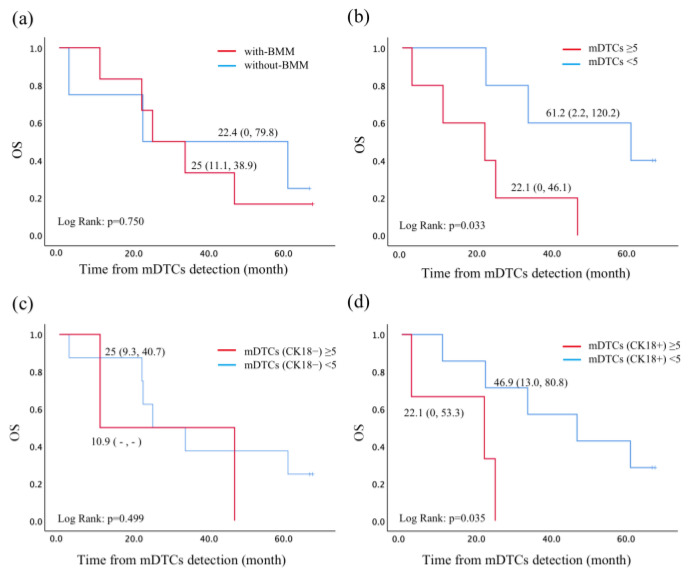
Survival analysis of breast cancer patients. (**a**) Kaplan–Meier curves for overall survival (OS) between breast cancer patients, with or without bone metastases by imaging examination. The difference in OS between patients stratified by mDTCs ≥ 5 and <5 (**b**), mDTCs (CK18^−^) ≥ 5 and <5 (**c**), and mDTCs (CK18^+^) ≥ 5 and <5 (**d**). The numbers beside the curve represent the median survival time and 95% confidence interval. BMM: bone marrow metastasis by imaging.

To investigate the clinical significance of mDTCs numbers, we conducted follow-ups for all breast cancer patients and recorded their accurate death times. Due to the small sample of prostate cancer patients, we only analyzed the prognosis of breast cancer patients. The median follow-up time of the 10 breast cancer patients was 29.4 months (range: 2.6–67.9 months), and the outcome was eight deaths and two losses. We divided the breast cancer samples into two groups, with or without bone metastasis, by imaging examination, and analyzed the OS difference. The result showed that the difference was statistically insignificant (*p* = 0.750, Figure 4a). Then we divided the sample into two groups according to the median level of mDTCs (median = 5 cells). The results showed that the OS of patients with mDTCs ≥5 and <5 was statistically different (*p* = 0.033, Figure 4b). Next, we performed a subtype analysis of mDTCs, and we found no difference in OS between patients with mDTCs (CK18^−^) ≥5 and < 5 (*p* = 0.499, Figure 4c), while mDTCs (CK18^+^) ≥5 and <5 showed a difference in OS (*p* = 0.035, Figure 4d). Similarly, we used the median and Q3 (3rd interquartile) of pCTCs for grouping breast cancer patients and we found no difference in OS (Figure S1). Collectively, the mDTCs level of patients with breast cancer is related to the patients’ OS. Patients with mDTCs levels ≥ 5 had a shorter OS than those with <5, especially for CK18^+^ mDTCs.

## 5. Discussion

Metastasis is the major cause of malignant tumor-related death. Different tissue-derived tumors have their preferential metastatic pathways and sites. Bone metastases are often observed in melanoma, and breast, prostate, bladder, lung, thyroid, and cervical cancers [7,17]. Bone metastasis causes insupportable pain and is very difficult to cure. Although metastasis is often accompanied by an advanced clinical stage, when or how metastasis occurs remains to be explored and answered. 

Clinically, bone metastasis is diagnosed based on obvious changes in morphology, structure, or metabolism. For example, on MRI imaging, an abnormal osteogenesis or osteo-breakage sign is associated with an unusually high level of glucose uptake when assessed using ^18^F-fluorodeoxyglucose labeled with positron emitters as tracers on PET-CT imaging, or abnormal absorption of radionuclide-labeled oleophilic ^99^mTc-methylene diphosphonate on bone scan imaging [18,19]. Although bone scintigraphy can detect bone metastases 3–6 months earlier than a regular X-ray [20], and the current spatial resolution of PET-CT can reach 1–3 mm, the sensitivity of both methods is still insufficient to find individual DTCs or DTM (disseminated tumor microemboli) [21]. Since DTCs or DTM are usually isolated cells or in the form of tiny cell clusters, it is very difficult to detect them using conventional imaging methods. It is worth mentioning that in addition to being scarce in number, another characteristic of DTCs is their dormancy status [11,22], which is undoubtedly a loophole in the current molecular imaging technology as PET-CT selectively identifies tumor cells with a high metabolism. Therefore, there is an urgent need for techniques that are more sensitive or not based on metabolic detection principles to detect those dormant DTCs or DTM.

According to recent reports, the dissemination of tumor cells might happen at an early stage, and they might stay in a dormant state for a long time, which has become an important hidden danger for metastasis and recurrence [22,23]. As a specific microenvironment, the bone marrow provides a natural shelter for DTCs or DTM to hide and survive. The window time between tumor cell spread (we call it the latent phase/dormant phase) and the formation of obvious metastasis (we call it the dominant metastasis phase) is varied. It has been reported that metastasis can appear 25 years post-surgery in breast cancer patients [11]. 

For a long time, researchers have been trying various methods to find bone metastasis as early as possible. This would help eliminate residual DTCs, improve the prognosis and prolong survival time. With a more in-depth understanding of the perniciousness of dormant DTCs, current strategies to wipe out dormant tumor cells mainly involve the following three aspects: (1) Keeping the cell dormant to prevent proliferation and relapse; (2) Activating dormant cells to increase drug sensitivity; and (3) Directly eradicating dormant cells [8,22]. Each of these three methods has its advantages and disadvantages, and clinicians can personalize the choice of treatment according to the different types and stages of tumor patients. Clinically, some postoperative adjuvant treatments are aimed at eradicating residual tumor cells in situ and in other sites of the body where mDTCs are hidden, such as the use of estrogen receptor antagonists to prevent the growth of hormone-dependent tumor cells. Abderrah et al. found that for ER^+^ breast cancer patients, extending the time of adjuvant anti-hormonal treatment from 5 years to 10 years could increase the patients’ survival rate [24]. However, despite the extensive use of postoperative adjuvant treatments, there are still many patients who suffer from recurrence or metastasis. Therefore, we believe that it is equally important to be able to technically detect and identify small residual foci of tumors as early as possible, as opposed to the treatment of tumors. 

Liquid biopsy has been considered a more sensitive method for the detection of tiny or residual tumor cells, compared with those conventional imaging methods or serum tumor markers. Liquid biopsy consists of a serum cell-free tumor DNA test (ctDNA), circulating tumor cell (CTC) detection, and serum exosome detection. As to CTCs’ detection, there are also many different methods such as flow cytometry, RT-PCR, and micro-fluidic chip-based methods [25]. In this study, we used SE-iFISH to detect CTCs in blood and DTCs in the bone marrow. The key points of SE-iFISH involve the enrichment and identification of tumor cells. The advantage of SE-iFISH is the combination of immunofluorescence staining and fluorescence in situ hybridization technology to maximize the findings of abnormal cells. In our previous study, we used EpCAM and CK18 to identify epithelium-derived tumor cells in the blood, and Her-2, CA-199, and αAFP for specific types of tumors such as breast, pancreatic, colon, or liver cancer [14]. Interestedly, no matter which marker had been used, positively stained tumor cells were rare to see, while CK18-positive cells seem to be more common than EpCAM-positive cells [14]. For this reason, we used CK18 as a tumor marker to identify epithelium-derived cells, which should not be present in the blood and bone marrow of a normal person. Another point to clarify is that four common channels of fluorescence microscopy were used (green for a tumor marker, orange for chromosome 8, blue for nuclei, and red for CD45 which is the marker of blood-derived karyocytes in blood and bone marrow). Thus, based on the limited channels, mesenchymal markers, such as Vimentin, or EMT markers, such as upregulated N-Cadherin, were not tested in this study. In our study, pCTC detection was performed using Cy5-labelled anti-Vimentin antibody to examine the expression of Vimentin associated with EMT, but we did not concomitantly detect both EpCAM and Vimentin or CK18 and Vimentin expressions in a single pCTC. Therefore, in most cases, EpCAM and Vimentin or CK18 and Vimentin expression was always exclusive. 

Taken together, the interesting findings of this study are that most CTCs in blood samples were CK18 negative, while most DTCs in the bone marrow were CK18 positive. These results are consistent with the consensus on EMT’s role in facilitating the shedding of tumor cells in and out of blood vessels, while also helping tumor cells adapt to the new environment and their colonization of metastatic sites after homing. The second valuable finding was that the detection of mDTCs by the SE-iFISH method compensates for the inadequacies and shortcomings of conventional imaging in the detection of bone micrometastases. Of course, the SE-iFISH method has its limitations. Unlike bone scans and PET-CT systemic examinations, SE-iFISH can only detect the metastasis of the sampling site, and therefore, there is the possibility of a missed diagnosis. In addition, the complexity and pain associated with bone marrow aspiration will also lead to poor patient acceptance. The third finding was that in addition to the positivity of the tumor marker, another characteristic of tumor cells is their chromosomal aneuploidy [26,27]. About 16% of the genes on chromosome 8 are related to tumors, and chromosome 8 aneuploidy is related to the occurrence of many types of tumors [16,28,29]. Since most CTCs and some DTCs did not express tumor markers, we used the CEP8 probe to detect aneuploidy in tumor cells and avoid undetected errors as much as possible. Our results confirmed that the copy number of chromosome 8 is also a good marker for pCTCs and mDTCs. Finally, our study indicated that CK18^+^ mDTCs are related to shorter overall survival time in breast cancer patients. Richter et al. used CK20 as a tumor marker and concluded that the number of CTCs has nothing to do with poor prognosis, but the number of DTCs in the bone marrow was related to the prognosis in patients with esophageal cancer, which is consistent with our observation [30]. However, the present study has several limitations. First of all, only 15 patients were included in this study, lacking the data support of a large sample size. Second, this was also a single-center analysis and lacked external validation. Finally, the SE-iFISH method only reflects the shortcomings of traditional imaging detection from the side, and the method itself has certain limitations, and it does not give enlightenment on better detection methods.

## 6. Conclusions

This study detected and analyzed mDTCs in breast and prostate cancer patients and revealed that the SE-iFISH method is more sensitive than conventional imaging methods for detecting the potential presence of micrometastases in bone marrow. Due to the small sample size of this study, it is necessary to conduct an in-depth analysis with a larger number of patients and multiple centers. In addition, due to some limitations of the SE-iFISH method, a more convenient, non-invasive, and multi-angle DTC detection method needs to be developed and studied.

## Figures and Tables

**Table 1:** General information of 10 breast cancer patients and 5 prostatic cancer patients.

Number	Gender	Age (yrs)	Stage	Sites of Bone Metastasis	Method Used for Bone Metastasis	Sites of Metastasis Other Than Bone	Treatment Ongoing	Treatment Duration
Breast cancer patients								
1	Female	59	IV	No	BS	Li, LN	Chemo	3 mths
2	Female	63	IV	Right first anterior rib (without ilium)	BS	Lu, LN, AF	Chemo	2 mths
3	Female	56	IV	Hip joint, lumbar spine, sacral spine, skull, humerus, shoulders, sternum, spine, bilateral ribs, bilateral femurs, and pelvic bones (possibly including ilium)	BS	No	Chemo + Endo	6 mths
4	Female	69	IV	Thoracic spine, ribs, lower lumbar spine, femur, right humerus, and pelvic bones (possibly including ilium)	BS	LN	Endo	3 mths
5	Female	56	IV	Ribs, sternum, lumbar vertebrae, and pelvic bones (possibly including ilium)	CT, MRI	No	Chemo	2 mths
6	Female	46	IV	No	BS	Lu	Before treatment	-
7	Female	61	IV	Multiple ribs, left sacroiliac joint, left ischium, left upper femur, and femoral head (without ilium)	BS	Lu, Li	Chemo + Target	3 mths
8	Female	60	IV	S1–3 vertebral bodies (without ilium)	PET-CT	Lu, Br, LN, Pl	Endo	1 year
9	Female	65	II	No	BS	No *	Endo	6 mths
10	Female	69	II	No	PET-CT	LN	Before-treat	-
Prostatic cancer patients								
11	Male	63	IV	Pelvis, femur, thoracic spine, ribs, and bilateral ilium	BS	No	Chemo	3 mths
12	Male	76	IV	Lower thoracic lumbar spine, sacrum, and bilateral ilium	BS	No	New diagnostic ^#^	-
13	Male	82	IV	Humerus, clavicle, ribs, sacroiliac joint, and ilium	BS	No	Endo	8 mths
14	Male	75	IV	L4-S3 vertebrae, scapula, femur, pelvis, and ilium	BS	LN	Endo	9 mths
15	Male	68	IV	Ribs, thoracic spine, sternum, and pelvic wall (without ilium)	CT, MRI	Li, LN, PC	Endo	4 mths

AF: armpit fat, BS: bone scan, Br: brain, Chemo: chemotherapy, Endo: endocrinotherapy, Li: liver, Lu: lung, LN: lymph node, PC: pelvic cavity, Pl: pleura. * Recurrence in chest wall, ^#^ New diagnosed in half a month.

**Table 2:** Characteristics of mDTCs, pCTCs and tumor tissue of 15 enrolled patients.

Number	mDTCs Number			pCTCs Number			Serum Tumor Markers								Primary Tumor Tissue			Metastatic Tissue		
	CK+	CK−	DTM	CK+	CK−	CTM	CEA	CA724	CA153	CA199	CA125	PSA	fPSA	fPSA/PSA	ER	PR	HER-2	ER	PR	HER-2
Breast cancer patients																				
1	5150	0	0	0	2	0	236.7	4.2	6.9	6.5	7.0	/	/	/	+	−	−	NA	NA	NA
2	0	149	4	0	7	0	1.4	7.1	14.4	4.5	21.4	/	/	/	−	−	−	−	+	+
3	14,370	0	0	0	7	0	3.3	14.5	22.3	24.6	29.7	/	/	/	−	+	−	NA	NA	NA
4	0	8	0	0	48	2	138.4	1.1	187.1	53.0	204.2	/	/	/	NA	NA	NA	+	+	+
5	21	0	1	13	209	14	24.0	/	113.7	19.6	26.9	/	/	/	+	+	−	−	−	−
6	0	0	0	0	29	1	0.9	0.8	8.9	2.8	27.6	/	/	/	−	−	+++	−	−	+++
7	0	0	0	0	0	0	253.0	1.9	257.4	22.7	597.0	/	/	/	++	++	+++	−	−	+++
8	0	0	0	0	4	0	7.8	2.6	11.1	27.9	252.7	/	/	/	+	−	+++	NA	NA	NA
9	2	0	0	0	5	1	0.9	2.0	10.7	9.2	6.5	/	/	/	NA	NA	NA	+	+	++
10	0	0	0	0	5	0	3.6	3.3	5.2	22.1	14.8	/	/	/	−	−	++	NA	NA	NA
Prostate cancer patients																				
11	642	0	0	0	45	3	4.6	74.1	37.5	0.8	11.7	151	20.1	13.3	/	/	/	/	/	/
12	50	0	18	0	2	0	2.2	4.7	15.0	10.2	11.8	81.8	13.3	16.3	/	/	/	/	/	/
13	11	35	5	3	34	0	3.0	8.0	/	25.3	6.2	108.	11.4	10.5	/	/	/	/	/	/
14	10	0	0	0	28	1	3.3	1.1	6.8	7.1	310.5	2.17	0.69	31.8	/	/	/	/	/	/
15	0	0	0	2	50	0	3.4	3.3	16.6	56.1	17.4	151	20.1	13.3	/	/	/	/	/	/

CTM: circulating tumor microemboli, CEA: carcinoma embryonic antigen, DTM: disseminated tumor microemboli, fPSA: free prostate-specific antigen, mDTCs: bone marrow disseminated tumor cells, NA not available, pCTCs: peripheral circulating tumor cells.

## Data Availability

All data generated or analyzed during this study are included in this published article [and its Supplementary Information files].

## Supplementary Materials

The following supporting information can be downloaded at: https://www.mdpi.com/article/10.3390/diagnostics14151629/s1, Figure S1: Kaplan-Meier curves for overall survival (OS) of breast cancer patients; Table S1: Ploidy number of chromosome 8 in mDTCs and pCTCs of 15 enrolled patients.

## List of Abbreviations

DTCs, disseminated tumor cells; mDTCs, bone marrow DTCs; pCTCs, peripheral circulating tumor cells; FISH, fluorescence in situ hybridization; DTM, disseminated tumor microemboli; CTM, circulating tumor microemboli; OS, overall survival; EMT, epithelial-to-mesenchymal transition; MET, mesenchymal-to-epithelial transition; ctDNA, cell-free tumor DNA.
