# Supplementary material for: Bone Marrow Disseminated Tumor Cell Detection Is Beneficial for the Early Finding of Bone Metastasis and Prognosis
Source: Diagnostics (Basel). 2024 Jul 29;14(15):1629. doi: 10.3390/diagnostics14151629 (PMC11311593; doi:10.3390/diagnostics14151629)
Supplement: Supplementary file 1 [file diagnostics-14-01629-s001.zip › Supplementary Material.pdf]

SE-iFISH method: To avoid contamination with epithelial cells, after discarding the first 2 ml of liquid, 6 ml of peripheral blood and 3 ml of bone marrow aspirate were collected using hCTC dedicated blood collection tubes. Immediately after collection, the blood collection tubes were mixed upside down 8 times, stored in the dark at room temperature, and used for detection and analysis within 24 hours. First, centrifugation was performed at room temperature for 15 minutes (200 g) and the supernatant to 5 mm above the pellet was removed, then 1× CRC wash solution was added to 6 ml and mixed by inversion. A total of 3 ml of sample density separation solution was added to the 50 ml centrifuge tube A and an electric pipette was used to slowly add the blood sample along the wall of tube A to the top layer of the separation solution, and the tube was centrifuged at room temperature for 6 minutes (350 g). Three layers of liquid were visible after centrifugation and the supernatant was transferred to a new 50 ml centrifuge tube (tube B). A total of 300 μl magnetic beads buffer was added to each tube which was shaken at room temperature for 20 minutes at 125 rpm in a shaker. Tube B was placed on a magnetic stand (Cytelligen, San Diego, U.S.A), and after 2 minutes, a pipette tip was used to transfer the liquid to a 50 ml centrifuge tube (Tube C) along the center of tube B. A 1×CRC washing solution was added twice for washing and then the tube was centrifuged at room temperature for 5 minutes (500 g) each time, and the supernatant was discarded to 100 μl. A total of 2 μl of antibody staining solution was added, mixed, and shaken in the dark for 10 min. Pre-prepared antibodies (CD45, CK18) were added and incubated in the dark for 20 min. All the liquid in centrifuge tube C was transferred to a 15 ml centrifuge tube, which was filled up with 1×CRC washing solution to 14 ml and centrifuged at room temperature for 5 minutes (500 g). After centrifugation, the supernatant was discarded to 100 μl and 100 μl of fixative was added and smeared before overnight incubation at 32-33 ℃ incubator to let it dry. The slides were taken the next day and 20 μl FR1 and 180 μl FR2 were added to each slide followed by fixation for 10 minutes. The surface liquid was removed with a vacuum pump, and the slides were rinsed 3 times with FR3 with absolute ethanol to remove FR3 before insertion into a cylinder for 2 minutes soaking. The glass slides were taken out, and dried with a hair dryer, and 8 μl of CEP8 probe was added, then each slide was

covered with a coverslip and sealed with glue before hybridization for 3 h in a hybridizer. After hybridization, each slide was washed three times with 150 μl of antibody washing solution, dried with a hair dryer, mounted with DAPI, scanned with a fluorescence microscope, and the images were subsequently analyzed.
