# Supplementary material for: Bone Marrow Disseminated Tumor Cell Detection Is Beneficial for the Early Finding of Bone Metastasis and Prognosis
Source: Diagnostics (Basel). 2024 Jul 29;14(15):1629. doi: 10.3390/diagnostics14151629 (PMC11311593; doi:10.3390/diagnostics14151629)
Supplement: Supplementary file 1 [file diagnostics-14-01629-s001.zip › Table S1.pdf]

Table S1 Ploidy number of chromosome 8 in mDTCs and pCTCs of 15 enrolled patients

Number	mDTCs Total number	mDTCs (CK-)			mDTCs (CK+)				pCTCs Total number	pCTCs (CK-)			pCTCs (CK+)			
		Tri-ploid	Tetra-ploid	Multi-ploid	Di-ploid	Tri-ploid	Tetra-ploid	Multi-ploid		Tri-ploid	Tetra-ploid	Multi-ploid	Di-ploid	Tri-ploid	Tetra-ploid	Multi-ploid
Breast cancer patients																
1	5150	0	0	0	5150	0	0	0	2	1	1	0	0	0	0	0
2	149	111	26	12	0	0	0	0	7	3	2	2	0	0	0	0
3	14,370	0	0	0	14,370	0	0	0	7	0	3	4	0	0	0	0
4	8	2	2	4	0	0	0	0	48	18	1	29	0	0	0	0
5	21	0	0	0	13	6	0	2	222	124	48	37	10	1	2	0
6	0	0	0	0	0	0	0	0	29	14	5	10	0	0	0	0
7	0	0	0	0	0	0	0	0	0	0	0	0	0	0	0	0
8	0	0	0	0	0	0	0	0	4	3	0	1	0	0	0	0
9	2	0	0	0	2	0	0	0	5	4	0	1	0	0	0	0
10	0	0	0	0	0	0	0	0	5	0	2	3	0	0	0	0
Prostate cancer patients																
11	642	0	0	0	642	0	0	0	45	15	8	22	0	0	0	0
12	50	0	0	0	50	0	0	0	2	2	0	0	0	0	0	0
13	46	29	4	4	7	2	0	0	37	19	7	8	2	1	0	0
14	10	0	0	0	10	0	0	0	28	20	2	6	0	0	0	0
15	0	0	0	0	0	0	0	0	52	25	12	13	1	1	0	0

mDTCs: bone marrow disseminated tumor cells, pCTCs: peripheral circulating tumor cells.
