# Supplementary figures and images for: Bone Marrow Disseminated Tumor Cell Detection Is Beneficial for the Early Finding of Bone Metastasis and Prognosis
Source: Diagnostics (Basel). 2024 Jul 29;14(15):1629. doi: 10.3390/diagnostics14151629 (PMC11311593; doi:10.3390/diagnostics14151629)

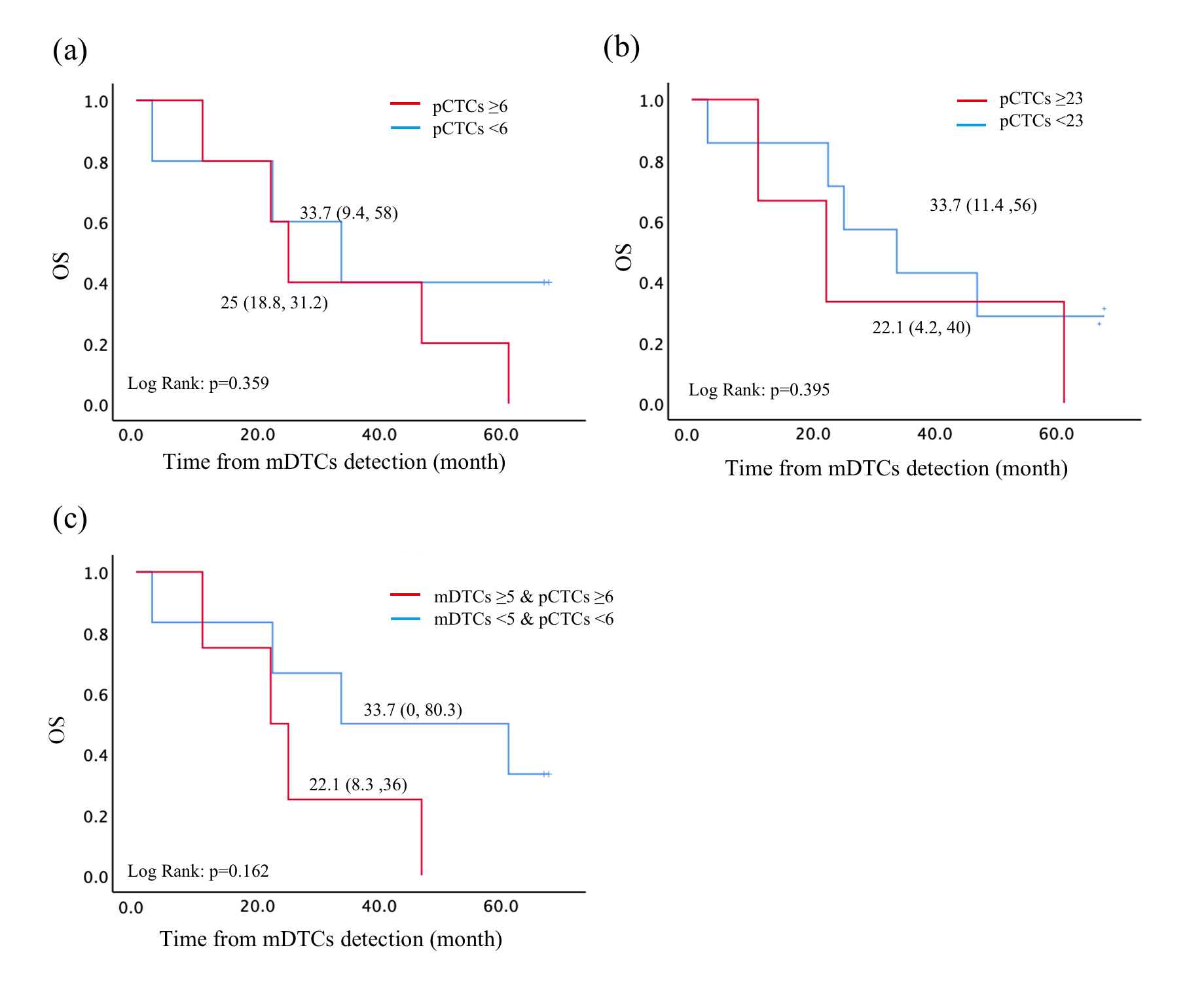

Supplement: Supplementary file 1 [file diagnostics-14-01629-s001.zip › Figure S1.tif]
